# Correlation between Serum Albumin Level and Severity of Acute Ischemic Stroke: A Cross-Sectional Study

**DOI:** 10.31729/jnma.8792

**Published:** 2024-10-31

**Authors:** Binod Pantha, Milan Khadka, Lochan Karki, Parimal Koirala

**Affiliations:** 1 Department of Medicine, National Academy of Medical Sciences, Bir Hospital, Kathmandu, Nepal

**Keywords:** *acute ischemic stroke*, *albumin*, *national institute of health stroke scale*, *modified rankin scale*

## Abstract

**Introduction:**

Stroke is a leading cause of disability with devastating consequences. Serum albumin has emerged as a significant prognostic marker in various conditions, including stroke. This study aims to investigate the relationship between serum albumin levels and stroke severity and outcomes in patients with acute ischemic stroke (AIS).

**Methods:**

An observational, cross-sectional study was conducted over six months at Bir Hospital, among patients admitted in medicine ward after obtaining the ethical approval reference number 34/20081/82. Total population sampling was done. Serum albumin at the time of diagnosis was measured. Ischemic stroke severity was scored based on NIHSS score on admission and the functional outcome was evaluated as modified Rankin Scale (mRS). Finally, correlation of serum albumin with the severity and the outcome of stroke (mRS) was made.

**Results:**

The study of 37 patients showed significant correlation between serum albumin and NIHSS score. The study showed moderate to strong negative correlation between serum albumin levels and mRS scores. The mean serum albumin level was 3.5±0.40 mg/dl.

**Conclusions:**

Significant correlation of serum albumin level with clinical severity at the time of admission and with the outcome during discharge was found. Thus serum albumin has prognostic significance in acute ischemic stroke.

## INTRODUCTION

Stroke is a leading cause of disability, with severe and widespread impacts.^1^ Serum albumin has emerged as a significant prognostic marker in various conditions, including stroke, with research indicating its association with both stroke severity and patient outcomes.^2,3^ The National Institute of Health Stroke Scale (NIHSS) is widely recognized for assessing initial stroke severity and predicting outcomes.^4-7^ The modified Rankin Scale (mRS) is used to evaluate functional disability, focusing on mobility and overall disability.^8-10^ Regardless of chosen cutoff point, serum albumin level remained an independent predictor of stroke outcome.^3,11^

Studies have established a baseline NIHSS score with severity and mRS with the functional outcome of stroke, which describes global disability with a focus on mobility.^12-14^

This study aims to investigate prognostic value of serum albumin levels in acute ischemic stroke, examining the relationship with NIHSS scores and mRS outcomes. By clarifying this, the study seeks to enhance understanding of serum albumin as a potential marker for stroke prognosis.

## METHODS

This is an observational cross-sectional study of six-month duration conducted at Bir Hospital in patients admitted in medicine wards. The study was done after getting ethical approval from the Institutional Review Committee (IRC) with reference number 34/20081/82. In this study, all adults aged 40 years or older presenting with history and clinical features suggestive of recent acute ischemic stroke admitted in the medicine wards in Bir Hospital within a study period were included. Total population sampling was done. Patients of age 40 years or older and both sexes with first-time ischemic stroke within 72 hours by clinical examination with ischemic lesion confirmed by CT Scan/MRI scan brain who gave informed consent from the patient or patient party were included. Patients with hemorrhagic stroke, ischemic stroke presenting after 72 hours of onset with recent severe infections, nephropathy, abnormal urinalysis, major trauma or surgery, major cardiac, hepatic, endocrinological disorders, skeletal disorders and cancer were excluded.

The National Institute of Health Stroke Scale Score (NIHSS) of all the patients at admission was calculated. serum albumin was sent as per the protocol. The correlation of NIHSS with admission serum albumin was calculated. The functional outcome was evaluated by a modified Rankin Scale (mRS)^13^ on discharge and its correlation value with admission serum albumin was calculated. At the time of discharge, the functional outcome was evaluated as. Data was entered in Microsoft Excel and descriptive analysis was performed.

## RESULTS

A total of 37 patients with Ischemic Stroke were enrolled in this study. Out of 37 patients, 20 (54.05%) patients were male and 17 (45.95%) were female. (Table 1). The mean age of the patients is approximately 67.74 ±11.32 years. The average serum albumin level was 3.5±0.40 mg/dl. In this study population with acute ischemic stroke, 30 (81.08%) had serum albumin in the range between 3-3.9 mg/dl (Table 1).

**Table 1:** Distribution of gender at various albumin level in patient with acute ischemic stroke (n=37)

Albumin (g/dl)	Female n (%)	Male n (%)	Total n (%)
<2.5	1 (2.70)	-	1 (2.70)
2.5-2.9	1 (2.70)	-	1 (2.70)
3-3.9	12 (32.43)	18 (48.64)	30 (81.08)
4-4.4	3 (8.10)	2 (5.40)	5 (13.51)
≥4.5	-	-	-
Total	17 (45.95)	20 (54.05)	37 (100)

The NIHSS score of 21-42 at admission was observed in 1 (2.70%) of the patients with acute ischemic stroke, Similarly, 29 (78.37%) of patients had NIHSS scores between the range 5-15 (Table 2).

**Table 2:** Distribution of gender at various NIHSS score (admission) in patient with acute ischemic stroke (n=37)

NIHSS	Female n (%)	Male n (%)	Total n (%)
0	-	-	-
1-4	2 (5.40)	1 (2.70)	3 (8.10)
5-15	12 (32.43)	17 (45.95)	29 (78.37)
16-20	2 (5.40)	2 (5.40)	4 (10.80)
21-42	1 (2.70)	-	1 (2.70)
**Total**	**17 (45.95)**	**20 (54.05)**	**37 (100)**

**Table 2:**
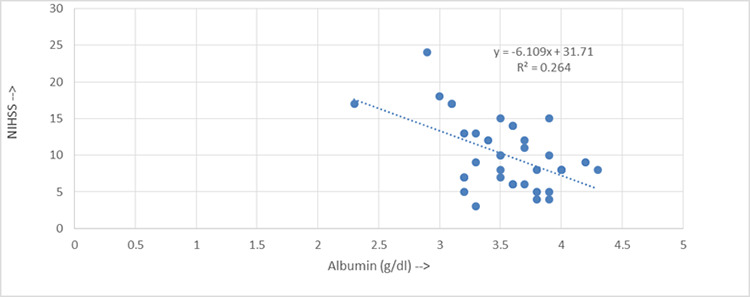
Distribution of gender at various NIHSS score (admission) in patient with acute ischemic stroke (n=37)

A Pearson correlation analysis showed a moderate to strong negative correlation between serum albumin levels and mRS scores (r = -0.530, p = 0.003).

A post-hoc power analysis was conducted to assess the statistical power of the observed correlations. For the correlation between serum albumin levels and NIHSS scores (r = -0.403), the power of the study was approximately 0.76, which is slightly below the conventionally desired power of 0.80. Similarly, for the correlation between serum albumin and the modified Rankin Scale (r = -0.530), the power of the study was higher, at approximately 0.90.

## DISCUSSION

In our study, most patients (81%) had serum albumin levels between 3-3.9 g/dl, which aligns with findings from similar research. For instance, Mahmud et al. observed that in a cohort of 50 acute ischemic stroke patients, the mean serum albumin level was 3.73±0.63 g/dl.^15^ Their study found an inverse relationship between serum albumin levels and both stroke severity (NIHSS) and functional outcomes (mRS), indicating that lower albumin levels are linked to worse outcomes, reinforcing its prognostic role in stroke patients. Similarly, Kasundra et al. reported that 28% of patients had serum albumin between 3-3.4 g/dl, while 30% had levels between 3.5-3.9 g/dl.^16^

Our study reported a mean NIHSS score of 10.08 at admission, indicating moderate stroke severity. In contrast, Sandeep et al. found a mean NIHSS score of 15.85±9.56 for survivors and 36.51±4.19 for those who did not survive, reflecting a cohort with more severe strokes.^17^ Notably, only 2.7% of our patients had an NIHSS score between 21-42, representing severe stroke, compared to the Cincinnati/Northern Kentucky Stroke Study, which reported a median NIHSS score of 3, indicating a prevalence of milder strokes in their cohort. These differences might be attributed to the varying patient populations and healthcare settings across the studies.^18^

In terms of functional outcomes, our study had a mean mRS score of 2.16±1.21 at discharge, suggesting relatively mild to moderate disability. This contrasts with Sandeep et al., where the mean mRS score was 5.13±0.3 in patients who died and 3.25±1.12 in survivors. Moreover, our study's mortality rate was significantly lower (2.7%) compared to 9.6% in Sandeep et al.'s cohort, possibly due to differences in patient demographics, stroke severity, or healthcare management practices.^17^

Kasundra et al. (2014) utilized the Barthel Index to measure functional independence one week after admission, focusing on short-term functional recovery and reported that higher serum albumin levels were associated with lower NIHSS scores at admission (p < 0.001) and better functional outcomes on the Barthel Index after one week (p < 0.001).^16^ Lower albumin levels correlated with higher NIHSS scores and poorer functional outcomes. Both studies confirm that lower serum albumin levels are associated with more severe stroke (higher NIHSS scores) and worse functional outcomes, supporting albumin's role as a prognostic biomarker in acute ischemic stroke.

In T. Dziedzic et al study, hypoalbuminemia, serum albumin level of 35 g/l, was found in 321 from 705 patients (45.5%). Which was similar result as in our study. In our study 45.9% (17 cases) had serum albumin of less than 3.5mg/dl.^11^ Our study had smaller sample size (37 patients), Dziedzic et al. (2007) had larger sample size (705 patients) with albumin levels measured within 36 hours of stroke onset, focusing on the prevalence of hypoalbuminemia and its immediate correlations with stroke severity and inflammatory markers. They used Scandinavian Stroke Scale and various serum protein fractions to assess the severity and inflammatory status shortly after stroke onset. Both studies find that lower serum albumin levels are associated with more severe strokes. Our study links lower albumin levels with higher NIHSS scores and poorer mRS outcomes, while Dziedzic et al. link hypoalbuminemia with more severe strokes and various inflammatory markers.

Idicula et al. (2009) examined the association of serum albumin levels with outcome and mortality in ischemic stroke patients, exploring the potential neuroprotective effect of albumin among 444 patients with ischemic stroke.^18^ They found that higher serum albumin levels were independently associated with better outcomes (OR = 1.12, p = 0.001) and lower mortality (OR = 0.88, p < 0.0001) after adjusting for age, sex, and NIHSS score at admission. Both studies find that serum albumin levels are significantly associated with stroke outcomes. Our study highlights the predictive value of albumin for functional outcomes and severity at discharge, while Idicula et al. demonstrate that high albumin levels are associated with better outcomes and lower mortality over a longer follow-up period.

Sandeep et al. found that serum albumin was a significant predictor of both short-term and long-term outcomes in stroke patients, suggesting beneficial mechanisms similar to those identified in other studies.^17^ They evaluated the prognostic significance of serum albumin in AIS, comparing it with NIHSS and mRS scores in an Observational study with a larger sample size of 135 patients, conducted at Liaquat University of Medical and Health Sciences. They reported a stronger negative correlation between serum albumin levels and both NIHSS (r = -0.724) and mRS scores (r = -0.774), with high specificity and sensitivity values in ROC curve analysis. Our study presents valuable insights with a smaller sample size, while Sandeep et al. provides a more comprehensive analysis with a larger sample, detailed statistical measures, and discussions on the mechanisms behind the observed correlations. Both studies agree on the potential of serum albumin as a useful biomarker in predicting stroke severity and outcomes, though Sandeep et al. offers more robust statistical evidence and broader implications.

Dash et al. (2020) explores the role of serum albumin as a prognostic marker in AIS, assessing its correlation with stroke severity (NIHSS) and outcomes at 1 week and 3 months (mRS) with a sample Size of 100 patients (58% male, 42% female; mean age 65±8.160 years).^19^ Our study is cross-sectional with outcome measures focused on discharge, while Dash et al. (2020) is prospective with extended follow-up, potentially allowing for the observation of trends over time. Both studies underscore the prognostic significance of serum albumin in AIS, with lower levels associated with worse outcomes. Both use NIHSS and mRS as primary measures of stroke severity and functional outcomes, yet the extended follow-up and larger cohort in Dash et al. (2020) offer additional insights into the long-term predictive value of serum albumin.

Nair et al. conducted a prospective observational study with 101 patients, focusing on those admitted within seven days of AIS. Stroke severity was measured using the NIHSS at admission, and functional outcomes were assessed using the Barthel index on the seventh day and third month post-stroke.^20^ They identified hypoalbuminemia in 18.8% of patients. They employed unpaired t-tests to compare serum albumin levels between different dependency groups as defined by the Barthel index, both on the seventh day and third month after stroke. They found that serum albumin levels were significantly higher in patients with moderate or slight dependency compared to those with total or severe dependency at the third month post-stroke (p = 0.008). Similar to our study, they concluded that lower serum albumin levels were associated with greater functional dependency at three months post-stroke, though the association was not significant at the seventh day.

A large-scale cohort study using data from the Third China National Stroke Registry (CNSR-III) by Zhou et al. (2020) included 13,618 patients with acute ischemic stroke (AIS) or transient ischemic attack (TIA) and utilized multiple logistic regression models and Cox regression models to assess the association between serum albumin levels and clinical outcomes.^3^ They concluded that low serum albumin levels are independently predictive of poor functional outcomes and mortality in patients with AIS and TIA. This provided robust evidence due to the large sample size and long-term follow-up, reinforcing the role of serum albumin as a critical prognostic factor.

A retrospective observational study involving 105 patients hospitalized after an acute stroke event by Kisialiou et al. (2022) examined a broader range of biomarkers, including ESR, fibrinogen, platelets, triglycerides, and albumin while investigating the association between these biomarkers and the size and location of ischemic lesions they found that higher albumin levels were correlated with smaller infarct sizes, suggesting a protective role, while other biomarkers were associated with different clinical outcomes.^21^ Both studies found that higher serum albumin levels are associated with better outcomes in stroke patients, suggesting its potential as a protective factor and prognostic biomarker in acute ischemic stroke. Our study supports the existing evidence, indicating that serum albumin is an independent predictor of functional outcomes in acute ischemic stroke. The consistency of our results with previous studies reinforces the need for further research into the underlying mechanisms through which serum albumin influences stroke outcomes.

One limitation of this study is the sample size of 37 patients, which may be slightly smaller than ideal for detecting moderate correlations, especially between serum albumin levels and NIHSS scores. Based on our sample size calculation, a larger cohort, particularly for the NIHSS correlation, may have provided more robust results. This limitation suggests that the study's power to fully detect moderate correlations could be impacted, and as such, the findings should be interpreted with this consideration in mind. Despite this, the significant results observed in this study are consistent with previously published research, indicating that serum albumin levels may play a significant role in predicting stroke severity and outcomes. Future studies with larger sample sizes are recommended to confirm these findings and strengthen the statistical power. Since ischemic stroke is a condition with long-term effects and the functional outcome changes with time, lack of follow up data may weaken clinical applicability. So future studies with longitudinal study design with larger sample size should be done.

## CONCLUSIONS

A moderate negative correlation was observed between serum albumin and NIHSS score while strong to moderate negative correlation was observed between serum albumin and mRS score.
